# ORGANIZATIONAL CHARACTERISTICS OF HIGHLY PERSON-CENTERED UNITS IN SWEDISH NURSING HOME

**DOI:** 10.1093/geroni/igz038.2577

**Published:** 2019-11-08

**Authors:** Anders B Sköldunger, Annica Backman

**Affiliations:** 1 Department of Nuring, Umeå, Sweden; 2 Departement of Nursing Umea University, Umea, Sweden

## Abstract

The movement from an institutional model of care towards a person-centred care as the gold standard of practice is now guiding the provision of care services in nursing homes around the world. The organizational context of care has been described as a determining factor for the extent to which staff can offer person-centred care. However, few studies have empirically investigated which factors that defines nursing home units as being person-centred. Providing information about organizational characteristics would therefor provide insight into an organizational context with capacity to enhance a person-centred care. Thus, the aim was to explore factors of nursing homes with high vs. low person-centred care with focus on organizational variables. The study was based on a cross-sectional national survey, and data on 4831 residents, 3605 staff, and facility variables were collected in 2014. Descriptive statistics and regression modelling were used to analyze the data. The preliminary results showed that characteristics of highly person-centred units were; dementia specific units and units with fewer number of beds. No significant differences were seen between private and public nursing homes in terms of degree of person-centred care. Person-centred units was characterized by managers supporting staff to provide individualized care based on the resident’s needs, as well as staff receiving supervision of a reg. nurse in the direct care. These findings can be seen as facilitators ’ for person-centred care, suggesting several contextual and organizational elements of significance for enhancing person-centred practice.

